# Role of Oxidative Stress in the Pathogenesis of Amyotrophic Lateral Sclerosis: Antioxidant Metalloenzymes and Therapeutic Strategies

**DOI:** 10.3390/biom11030437

**Published:** 2021-03-16

**Authors:** Pavlína Hemerková, Martin Vališ

**Affiliations:** Department of Neurology, Charles University, Faculty of Medicine and University Hospital Hradec Kralove, 500 05 Hradec Kralove, Czech Republic; valismar@fnhk.cz

**Keywords:** amyotrophic lateral sclerosis, superoxide dismutase, catalase, glutathione peroxidase

## Abstract

Amyotrophic lateral sclerosis (ALS) affects motor neurons in the cerebral cortex, brainstem and spinal cord and leads to death due to respiratory failure within three to five years. Although the clinical symptoms of this disease were first described in 1869 and it is the most common motor neuron disease and the most common neurodegenerative disease in middle-aged individuals, the exact etiopathogenesis of ALS remains unclear and it remains incurable. However, free oxygen radicals (i.e., molecules containing one or more free electrons) are known to contribute to the pathogenesis of this disease as they very readily bind intracellular structures, leading to functional impairment. Antioxidant enzymes, which are often metalloenzymes, inactivate free oxygen radicals by converting them into a less harmful substance. One of the most important antioxidant enzymes is Cu^2+^Zn^2+^ superoxide dismutase (SOD1), which is mutated in 20% of cases of the familial form of ALS (fALS) and up to 7% of sporadic ALS (sALS) cases. In addition, the proper functioning of catalase and glutathione peroxidase (GPx) is essential for antioxidant protection. In this review article, we focus on the mechanisms through which these enzymes are involved in the antioxidant response to oxidative stress and thus the pathogenesis of ALS and their potential as therapeutic targets.

## 1. Introduction

Although the clinical symptoms of amyotrophic lateral sclerosis (ALS) were first described by French neurologist Jean-Martin Charcot in 1869, this disease remains fatal. Because ALS causes the progressive degeneration of motor neurons in the brain stem, as well as the cerebral cortex and spinal cord, death due to respiratory failure occurs within three to five years [1,2]. ALS is the most common motor neuron disease. Its mean incidence rate is 2.8/100,000 in Europe and 1.8/100,000 in North America. Its prevalence is 5.4/100,000 in Europe and 3.4/100,000 in North America [3]. The degeneration of upper motor neurons, which conduct impulses from the cortex and brainstem to lower motor neurons through corticospinal and corticobulbar tracts in the healthy brain, in ALS leads to spasticity, overall clumsiness, markedly vivid reflexes and pyramidal signs. The degeneration of the lower motor neurons in the ventral horn of the spinal cord and nerve roots, which conduct impulses to innervated muscle fibres, leads to muscle weakness, atrophy, spasm and fasciculations. Patients usually present with a combination of symptoms due to the impairment of both types of motor neurons. Atypical forms of the disease occur when impairment of either the upper or lower motor neurons is highly predominant.

Despite its profound impact on motor neurons, ALS has additional effects. For example, neuronal loss in the frontotemporal region, resulting in the symptoms of dementia, is common in ALS, and approximately 15% of patients with frontotemporal dementia (FTD) develop ALS as well, and 5–22% of patients with ALS also develop FTD. Furthermore, FTD-type cognitive dysfunction is observed in almost 50% of patients with ALS [4,5]. Patients with motor neuron diseases have also been reported to have symptoms of extrapyramidal system damage, as confirmed by subsequent neuropathological assessments. For example, Pradat et al. published on a patient with typical dysfunction of both the upper and lower motor neurons, indicating ALS, who developed chorea after a disease duration of 10 years [6,7]. Moreover, metabolic disorders, typically hypermetabolism associated with weight loss and hyperlipidaemia, are often associated with ALS, indicating the probable involvement of the hypothalamus in the development and progression of ALS [8].

### 1.1. Oxidative Stress

Oxidative stress is typically defined as an imbalance in the production of free oxygen radicals or reactive oxygen species (ROS), i.e., molecules with one or more free electrons that readily bind intracellular macromolecules, impairing the function of these structures, and antioxidant protection in the body. Such molecules (free radicals and ROS) are ubiquitously produced in living organisms. The mitochondrial wall contains the respiratory chain, which consumes 85–90% of the total oxygen consumed by the body; however, given that electrons escape from the respiratory chain and molecular oxygen is incompletely reduced during aerobic metabolism, mitochondria also produce the highest levels of ROS in the cell [9]. Oxidative enzymes, such as cytochrome P450 in the endoplasmic reticulum and NADPH oxidase, which is responsible for the production of superoxide in phagocytes during pathogen destruction among other functions, are somewhat less involved in the production of ROS [10]. Exposure to ionizing radiation (through the interaction of radiation with water) and cellular exposure to hypoxia/hyperoxia also lead to ROS production [9,11].

Despite the damaging effects of ROS on the body, not all of their effects on the body are negative. For example, through easily oxidizable intracellular groups, so-called redox sensors, ROS activate protein kinase and transcription factor signalling cascades and thus activate or inhibit many cellular processes, such as proliferation, differentiation, metabolism, and apoptosis. Kelch-like ECH-associated protein 1 (Keap1), nuclear factor erythroid 2-related factor 2 (Nrf2), antioxidant response element (ARE), PI3K-Akt, NF-κB or MAPKs, for example, function as ROS signalling molecules in these cellular pathways [12]. Specifically, ROS are essential for the differentiation of adipocytes from bone marrow mesenchymal stem cells [13]. They are also involved in the sustained proliferation and differentiation of epidermal cells in the basement membrane region, which is involved in the constant renewal activity of the epidermis. [14]. When the level of ROS is too low, the regenerative capacity of neural and spermatogonial stem cells is reduced [15]. Furthermore, the beneficial effect of exercise with a stress load is mediated at least in part by ROS, and ROS also subsequently activate the NrF2/ARE signalling pathway and antioxidant protective mechanisms of an organism after exercise [16]. Furthermore, ROS are part of the immune response. Despite the involvement of ROS in the fight against microbes (bacteria and fungi; their antiviral effects are unclear), they also suppress specific immune responses and inflammatory reactions. Therefore, for example, patients with chronic granulomatous disease (CGD) and a mutation in the gene encoding the NOX 2 protein, a catalytically active superoxide-producing NADPH oxidase, lack phagocytes with bactericidal capacity and suffer from reduced immunity and recurrent purulent infections from an early age [17,18].

In addition to their role in vital cellular processes and the immune response, ROS play very important roles in learning and memory processes. Indeed, NOX enzymes are involved in glutamatergic neurotransmission and affect NMDA receptors [19]. The role of ROS in learning and memory has also been well demonstrated in patients with CGD. Almost a quarter of these patients have an IQ of 70 or less. This is thought to be because chronic diseases require repeated hospitalizations, as evidenced by the finding that in patients with cystic fibrosis who have approximately the same number of recurrent infections and hospitalizations, no reduction in cognition was observed [20,21]. In addition, cognitive deficits developed in CGD model mice even when the mice were kept in a non-pathogenic environment in which infections did not develop [22].

#### 1.1.1. Superoxide and Its Role in the Formation of Other ROS

**Figure 1:**
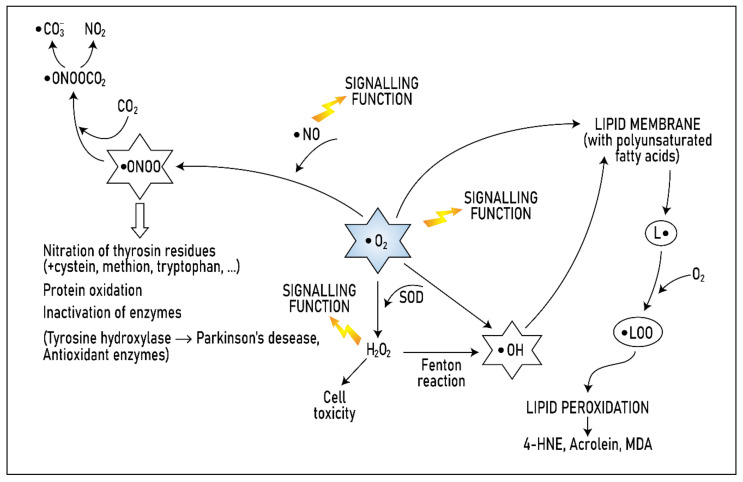
Role of superoxide in the formation of other radicals. Modified from [23].

Superoxide, the most common free-radical-containing molecule in the human body, is produced in the respiratory chain (when electrons escape and combine with oxygen) through the activity of NADPH oxidase and some other enzymes (xanthine oxidase, cyclooxygenase, and lipoxygenase), via the reaction of haemoglobin with oxygen or through the autoxidation of various substances (ascorbate, glutathione and other thiols and catecholamines). Although it contains a free radical, superoxide itself is only slightly reactive and therefore relatively harmless. It can undergo spontaneous conversion through so-dismutation to hydrogen peroxide, which is immediately eliminated by linked reactions catalysed by catalase and peroxidase. However, other highly harmful ROS, such as the hydroxyl radical, peroxynitrite and hypochlorous acid, can form from superoxide. The crucial role of superoxide in the production of other radicals is illustrated in Figure 1. Considering that the half-life of the hydroxyl radical is very short, there is no mechanism for its elimination, and organisms therefore must eliminate superoxide itself [23].

Among oxygen radicals, superoxide is relatively less reactive. Its danger lies in its ability to transform into much more dangerous radicals. This is because the hydroxyl radical (• OH), which is extremely reactive and reacts immediately with biomolecules in the environment, can form directly from superoxide.

In the presence of the enzyme SOD, superoxide is converted to hydrogen peroxide (H2O2). Although hydrogen peroxide is not a very reactive form of oxygen, when it interacts with a transition metal (copper or iron), the most dangerous radical in the human body, the hydroxyl radical (• OH), forms.

Superoxide can also react with nitrous oxide, a gaseous radical with a short biological half-life that can both enter the body from the external environment, for example, from smog, or be produced endogenously in the body, for example, as a vasodilator. During this reaction, peroxynitrite (• ONOO), which is responsible for nitration and hydroxylation of the amino acid tyrosine as well as also other amino acids, such as cysteine, methionine, and tryptophan, is formed.

The actions of peroxynitrite lead to the oxidation and nitration of proteins, lipid peroxidation, and the inactivation of many enzymes, such as antioxidants and tyrosine hydroxylase, which is necessary for the formation of dopamine, the activity of which is reduced in Parkinson’s disease.

Both superoxide and the hydroxyl radical act directly on lipid membranes, inducing lipid peroxidation. Unsaturated higher fatty acids, which comprise phospholipids in the membrane, are the main lipids subject to peroxidation. Free radicals cleave a hydrogen atom from these fatty acids, which forms a free radical on the fatty acid (• L). This changes the structure of the fatty acid, rendering it very reactive. Further reaction with oxygen produces a peroxyl radical (• LOO), which attacks a neighbouring fatty acid and removes a hydrogen atom from it, causing the radical species to become a lipoperoxide. In addition, the attacked fatty acid becomes a radical that then attacks other molecules. Thus, a chain reaction is initiated, resulting in changes in membrane integrity and permeability. In addition, the end products of lipid peroxidation can be mutagenic and carcinogenic, and some are markers of lipid peroxidation levels. For example, 4-hydroxynonenal (HNE) and malondialdehyde can form various covalent protein adducts, for example, by reacting with deoxyadenosine and deoxyguanosine in DNA, to generate advanced end products of lipoxidation.

#### 1.1.2. Oxidative Stress and Its Role in ALS Pathogenesis

All aerobic cells are exposed to oxidative stress; however, as neurons are particularly sensitive and susceptible to damage from such stress for several reasons, oxidative stress plays a critical role in ALS, in which motor neurons are rendered dysfunctional.

First, neurons are remarkably large cells, with a cell body up to 60 μm in diameter and an axon up to 1 m in length. The regenerative capacity of postmitotic neurons is low. The large metabolic demands due to the large size of neurons are associated with a high oxygen demand, resulting in increased ROS production [24,25,26].

In addition, motor neurons express relatively low levels of calcium-binding proteins. Thus, excess calcium ions tend to enter mitochondria, leading to increased ROS production [27,28]. Furthermore, the expression of some protective antioxidant proteins, such as catalase, is lower in motor neurons than in other cell types [29,30].

Indicators of oxidative damage have been repeatedly shown to be elevated in the spinal cord and motor cortex, mostly in large ventral horn motor neurons [31,32,33,34]. Higher levels of lipid oxidation markers were also detected in the motor neurons, astrocytes and microglial cells of patients with sALS than in those of healthy individuals [35,36]. Furthermore, HNE, a product of lipid peroxidation, has been found in both the sera and CSF of patients with ALS [37].

Elevated levels of oxidative stress trigger or exacerbate the neurodegenerative process in several ways. When cellular synapses (whether those of neurons or glia) are exposed to excessive ROS, binding of the major excitatory neurotransmitter glutamate to its transporters is reduced [38,39,40,41]. When the glutamate concentration in the synapse increases, glutamate receptors are excessively stimulated, leading to an increased concentration of calcium ions within motor neurons, which is harmful to the cell [42]. Calcium then enters the mitochondria, which leads to impaired mitochondrial function and thus the further production of ROS.

In a study by Spreux-Varoquaux, elevated glutamate levels were correlated with faster ALS progression [43]. Moreover, when neurons were exposed to the glutamate agonists AMPA or kainate, mitochondrial calcium uptake increased, followed by a subsequent increase in ROS production [29].

ROS released from damaged neurons act on surrounding glial synapses, where, as they do in neurons, they increase the concentration of glutamate and induce a consequent increase in calcium influx into the cell and mitochondria, and this again leads to impairment of glial function and increased ROS production. In addition, ROS can activate glia and thus induce the production of proinflammatory cytokines and other ROS. As a result, the process of neuronal degeneration, which may have initially been focal, spreads diffusely throughout the neuronal population [44,45].

ROS also negatively impact the neurocytoskeleton. Neurofilaments are an essential part of these structures in the body and the protrusions of neurons of the CNS and peripheral nervous system. Abnormal accumulation of neurofilaments is often found in patients with ALS, as well as in mouse models of the disease [46,47,48,49]. In addition to their other roles, ROS play a role in this accumulation of neurofilaments because ROS produced by mutant SOD primarily binds the NFL. As a result of this binding, the whole neurofilament triplet structure is disrupted and neurofilament aggregation occurs [50,51].

### 1.2. Genetic Risk Factors and Their Relationship to Oxidative Stress

The familial form of ALS (fALS), which is caused by autosomal, usually dominant and highly penetrant mutations, accounts for approximately 10–15% of ALS cases [52]. Compared to the sporadic form (sALS), which most commonly develops at the age of 58–63 years, fALS is characterized by a younger average age at disease onset [53,54,55].

#### 1.2.1. C9ORF72 Gene Mutation

A mutation in the *C9ORF72* gene on chromosome 9 is the most common genetic cause of ALS. The prevalence of this mutation is up to 40% in individuals with fALS and 7–10% in individuals with sALS, and this mutation is also common in patients with FTD [56,57,58]. The mutation involves a non-coding GGGGCC hexanucleotide repeat expansion in the *C9ORF72* gene, and three hypotheses for the pathogenic effect of the C9ORF72 gene mutation have been proposed. The first postulates that the amount of the normal gene product, i.e., the C9ORF72 protein, is reduced; however, the function of this protein is not yet known. Another hypothesis is that the mutated C9ORF72 protein carries out a new but toxic function in cells. Finally, ALS pathogenesis may involve the accumulation of dipeptide repeats resulting from non-ATG translation of the mutated *C9ORF72* gene. This is because this mutation in the C9ORF72 gene results in the production of five types of dipeptide repeat proteins, at least three of which, poly-PR, poly-GR and poly-GA, are toxic to neurons [59,60]. Gonzalez et al. showed that in C9ORF72-expressing neurons (grown from induced pluripotent stem cells, iPSCs), the poly-GR protein bound mitochondrial ribosomes, leading to impaired mitochondrial function and consequently increased levels of neuronal oxidative stress and in addition, when oxidative stress was pharmacologically or genetically suppressed in the examined cells, oxidative DNA damage was alleviated [61].

#### 1.2.2. SOD1 Gene Mutation

A mutation in the SOD1 gene, which encodes Cu2+/Zn2+ superoxide dismutase (SOD), the second most common mutation in ALS, is directly related to oxidative stress. The specific effects of *SOD1* mutation are described in detail later in this review.

#### 1.2.3. TARDBP Gene Mutation

A mutation in the *TARDBP* gene on chromosome 1, the gene product of which (TDP-43) binds DNA and RNA in the nucleus, playing a role in transcription in healthy neurons is the third most common mutation in ALS. This mutation, which is found in approximately 5% of patients with fALS and 1% of patients with sALS, causes the abnormal phosphorylation and accumulation of TDP-43 in the cytoplasm of motor neurons, resulting in the formation of inclusions [62,63,64,65,66,67]. Cytoplasmic aggregation of TDP-43 occurs both in patients with sALS and in patients with the C9ORF72 mutation described above [68]. While excess TDP-43 leads to cytoplasmic inclusions that impair cell function, depletion of the protein from the nucleus leads to the dysregulation of mRNA metabolism [69]. Because both the loss of the TDP-43 protein and its overproduction can lead to the development of disease, strict regulation of the expression of this protein is thus important.

Further showing the role of TDP-43 in oxidative stress, Iguchi et al. showed that oxidative stress induced by glutathione depletion led to changes in the C-terminal phosphorylation, fragmentation, insolubilization and cytoplasmic distribution of TDP-43, effects that have been seen in ALS as well as other neurodegenerative diseases. These changes were observed in both the NSC-34 cell line (the motoneuron-like *cell* line) and primary cortical neurons after exposure to ethacrynic acid (which can deplete glutathione) or hydrogen peroxide [70].

Additionally, Wang et al. suggested that the mutation of TDP-43 alone leads to increased ROS production. By using electron microscopy, the researchers analysed cellular and animal models of TDP-43 proteinopathy and detected morphological mitochondrial damage associated with increased ROS production [71].

#### 1.2.4. FUS Gene Mutation

The fourth most common cause of fALS in the European population (and the second most common cause of fALS in Asia) is a mutation in the *FUS* gene, which encodes the RNA-binding protein fused in sarcoma (FUS). This mutation is often associated with the juvenile and early-onset forms of ALS [72]. Like the mutation in *C9ORF72* discussed above, it is not entirely clear whether *FUS* mutation induces loss of function or, conversely, gain of a new, toxic function. Many of the functions of the FUS protein are shared by the TDP-43 protein, as both proteins are involved in gene expression, pre-mRNA splicing, RNA transport, and translation regulation. However, the proteins show specificity for different RNA sequences, and FUS is also involved in DNA restoration processes [73]. If the body’s protective antioxidant mechanism fails, DNA with single-strand breaks (DNA SSbs) accumulates in the CNS. The X-ray cross-complementing group 1 (XRCC1)/ligase III (Lig III) complex, in which the FUS protein is also involved, plays a crucial role in the restoration of these DNA SSbs [74].

Under normal conditions, the poly ADP ribose polymerase 1 (PARP1) protein first transiently binds the site of DNA damage, mostly likely serving as a signal for the initiation of DNA restoration that invokes other restorative proteins [75].

FUS is required for the abovementioned XRCC1/Lig III complex, which subsequently binds damaged DNA. Due to the specific interaction of FUS with DNA at the site of damage in vitro and its rapid and transient response to DNA damage in cells, FUS probably serves as a sensor of DNA breaks and as coordinator of the overall DNA restoration process [76,77]. Lig III functions to attach the restored chain by forming a phosphodiester bond. The interaction of Lig III with XRCC1, which protects it from degradation by the proteasome, is important for the stability of Lig III. This interaction also allows targeting of Lig III to the site of breakage [78,79].

Two types of *FUS* mutations can adversely affect DNA restoration in different ways. *FUS* with the P525L mutation is not found in the nucleus, where it is needed, but rather is located in the cytoplasm. With the R521H/C mutation, the formation of the reparative PARP1/XRCC1/Lig III complex fails. This results in the accumulation of SSbs, followed by the accumulation of double-strand breaks and acceleration of the neurodegenerative process [74].

In summary, it is generally still unclear whether oxidative stress and mitochondrial damage occur at the beginning of these pathological processes and then cause the impairment of RNA metabolism or if disordered RNA metabolism causes mitochondrial damage and oxidative stress is generally unclear [80]. In support of the first hypothesis, findings suggest that both the TDP-43 and FUS proteins tend to translocate to the cytoplasm to form inclusions upon exposure to excessive oxidative stress [81,82]. These phenomena are consistent with the frequent detection of these inclusions in the vicinity of stress bodies in both cell models and patients [83]. Stress bodies, formed when cells are exposed to stress, contain mRNA encoding common cellular proteins. However, during stress, the corresponding mRNAs are inactive, preventing damage. The function of stress bodies is therefore protective. However, under conditions of prolonged cellular stress, stress bodies also trap TDP-43 and FUS and participate in their aggregation and, consequently, the loss of their nuclear functions [82,84]. Thus, these data support the notion that oxidative stress and mitochondrial damage impair RNA metabolism. The second hypothesis, which suggests that mitochondrial damage and oxidative stress are the consequence of disordered RNA metabolism, is supported by several findings. Nuclear TPD-43 negatively regulates FOXO transcription factors, including the transcription factor FOXO3a, whose activation is necessary to downregulate the expression of many genes that encode proteins with mitochondrial functions. Thus, FOXO3 activation results in reductions in mitochondrial protein expression and activity of the mitochondrial respiratory chain. When *TARDBP* is mutated, the translated TDP-43 protein is transported out of the nucleus and thus cannot negatively regulate FOXO3. As a result, mitochondrial damage occurs [85,86].

Similarly, the FUS protein interacts with peroxisome proliferator-activated receptor-gamma coactivator (PGC)-1 α, a member of a family of transcription coactivators that plays a central role in the regulation of cellular energy metabolism and stimulates mitochondrial biogenesis. This interaction is necessary for the transcription of several genes involved in protection against oxidative stress, such as catalase and Mn-SOD. Thus, the mutation of FUS may adversely affect its interaction with PGC-1 alpha, leading to the reduced expression of protective antioxidant genes [87].

### 1.3. Non-Genetic Factors Associated with Increased Oxidative Stress Are Also Risk Factors for ALS

#### 1.3.1. Life-Style Factors

A number of lifestyle-related factors have been shown to increase the risk of ALS. Smoking is a risk factor for ALS in women, particularly postmenopausal women. However, this is not true in men, but the cause of this difference remains unclear [88,89,90]. Cigarette smoking generates a large amount of free radicals, such as superoxide and free hydroxyl radicals. Furthermore, intracellular antioxidant protection is impaired by cigarette smoking, and the levels of glutathione, ascorbic acid, glutathione, peroxidase and catalase, all of which are antioxidants, are reduced. As a result, protein and lipid oxidation and DNA damage are enhanced. In addition to its participation in lipid peroxidation, formaldehyde increases the levels of lead and cadmium, both of which accelerate the oxidative damage process, in the blood and seminal plasma [91,92].

Regarding dietary habits, the risk of ALS has shown an inverse relationship with the intake of antioxidants, such as vitamin E, and polyunsaturated fatty acids (PUFAs) (see below). Whether dietary glutamate, which in itself can induce oxidative stress, increases the risk of ALS is unclear [93].

Additionally, the role of physical activity in the pathogenesis of ALS also remains unclear. According to some studies, a low premorbid BMI is associated with an increased risk of ALS and higher mortality [94,95]. Several studies have shown that professional baseball and football players and other athletes who engage in a high level of physical activity have a higher risk of ALS than other individuals [96,97,98]. Interestingly, ROS production is likely to increase when high-intensity physical exertion occurs. Under conditions of high-intensity physical activity, decreased glutathione levels and increased levels of 8-oxo-2’-deoxyguanosine, an oxidized derivative of deoxyguanosine, a major product of DNA oxidation, were detected in lymphocytes [99,100] Another physical risk factor is CNS trauma, especially if the trauma is recurrent. The stress response to trauma involves the production of ROS and oxidative tissue damage [101]. Because such repeated head impacts, as well as doping and the chemicals used to treat injuries, may play a role in increasing the risk of ALS [96,102], it is unclear whether physical activity itself is related to an increased risk of ALS.

#### 1.3.2. Environmental Factors

In addition to genetic and lifestyle-related factors, exposure to lead is associated with an increased risk of ALS. Increased lead levels were observed in both the peripheral blood and bones of patients with ALS compared to those of healthy controls, although the release of lead from the bones into the periphery due to altered bone metabolism may contribute to this increased risk [103,104,105]. In neuroblastoma cell lines, lead exposure led to the depletion of glutathione, which is capable of preventing damage caused by reactive oxygen species. Astroglia, which generally protect neurons against oxidative stress under physiological conditions, also act as a buffer system for lead. However, astroglia themselves are negatively affected by this buffer function, as gradual morphological and functional changes in the glia eventually produce ROS [106,107].

Additionally, the neurotoxic properties of manganese and iron are also well known [108]. For example, welders exposed to manganese often develop motor function impairment, particularly fine motor skills [109,110,111]. Additionally, although iron acts as a co-factor for a number of important enzymes, it accumulates in nerve tissue in many neurodegenerative diseases, including ALS. Iron accumulation in the ventral horn and motor cortex, regions responsible for innervation of the hand, has been observed in patients with ALS, which is consistent with the initial clinical symptoms commonly observed in patients with ALS, namely problems with fine motor skills [112,113].

Another environmental factor associated with an increased risk of ALS is exposure to pesticides [114,115,116]. The active metabolites of most organophosphates used in agriculture, such as the insecticides chlorpyrifos, parathion and malathion, which reduce the level of glutathione in neurons and astroglia, deplete antioxidants [117,118,119]. Furthermore, when cultured lymphocytes were exposed to organophosphates, glutathione levels decreased, and the levels of malondialdehyde and 8-oxo-2′-deoxyguanosine, markers of oxidative damage, were increased [117,120]. Additionally, paraquat, a non-selective herbicide used mainly in soybean, maize and rice cultivation, depletes glutathione from the CNS. Furthermore, NADPH activates superoxide-producing oxidase in microglia and inhibits respiratory chain function, increasing the production of oxygen radicals [121,122,123,124,125,126,127,128].

Taking into account these environmental risk factors, it is no surprise that ALS is more common in individuals of certain professions involving exposure to chemicals, pesticides, heavy metals or electromagnetic fields, such as painters, those who work with leather or rubber, welders, gardeners, and electricians [129,130,131]. However, because a large part of the population is exposed to these risk factors, it remains unclear why only a small proportion of individuals with these professions develop the disease. Generally, the development of ALS requires both genetic and non-genetic factors. ALS does not always develop in carriers of mutations with high penetrance. For example, a set of monozygotic twins carrying a mutation in the C9ORF72 gene generating approximately the same number of repeats was reported. ALS developed in one twin who smoked and had a history of a head injury but did not develop in the other twin [132]. Knowledge of these non-genetic factors, which can be altered, might help members of families with a potential genetically increased risk of ALS learn how to modify their lifestyle to potentially decrease the likelihood of ALS.

## 2. Antioxidant Protection in the Body—Three Main Enzymes

Antioxidants such as polyphenols, ascorbic acid, vitamins A and E, glutathione, melatonin, coenzyme Q, beta-carotene, and alpha-tocopherols, as well as antioxidant enzymes, including SOD, catalase, glutathione peroxidases (GPxs), reductases and s-transferases, are involved in antioxidant protection of the body. Generally, antioxidants and antioxidant enzymes can be divided into three groups based on their activity [133]. First-line antioxidants prevent the formation of radicals by neutralizing molecules with the potential to become highly reactive radicals or by preventing the transformation of poorly reactive radicals into more reactive molecules. The three key enzymes in this group are SOD, catalase and GPx, but transferrin and ceruloplasmin are also members of this group [23]. Second-line antioxidants scavenge or neutralize radicals by acting as electron donors. This group of antioxidants includes ascorbic and uric acids, glutathione and vitamin E. Finally, third-line antioxidants repair damage caused by ROS. This group includes de novo-formed enzymes that repair DNA (polymerases and nucleases), damaged lipids and proteins. Third-line antioxidants also remove damaged molecules to prevent their aggregation and toxicity [23]. Below, we discuss the three key first-line antioxidant enzymes and their relationship with ALS.

### 2.1. Superoxide Dismutase, SOD

Present in all aerobic organisms, SOD is the most powerful cellular antioxidant. To function properly, SOD needs a metal atom as a co-factor; thus, SOD is classified as a metalloenzyme. Fe^2+^SOD, which is phylogenetically younger than other forms of SOD, is found in prokaryotes and the chloroplasts of some plants. Mn^2+^SOD1 is also predominantly found in prokaryotes but is also found in eukaryotic mitochondria. Finally, Cu^2+^/Zn^2+^SOD (SOD1) is found in mitochondria, peroxisomes, and the cytosol of the cells of the eukaryotes plants and animals [133,134]. SOD1 is particularly abundant in the cytosol and requires posttranslational modifications to become functional, including zinc-binding, copper delivery and disulfide bridge formation, resulting in a dimeric structure [135,136,137,138,139,140]. Each subunit of the dimeric mature enzyme includes one atom of zinc and one atom of copper [141,142]. While zinc stabilizes the SOD1 molecule and thus plays a structural role, copper is necessary for proper catalytic function of the enzyme and thus the scavenging of free radicals. The delivery of copper to SOD1 depends on the aptly named copper chaperone for SOD1 (CCS) [142,143,144].

A significant decrease in the enzymatic activity of SOD1 occurred in mice with genetic deletion of CCS [145,146]. CCS not only supplies copper to the enzyme but is also involved in and necessary for all three SOD1 posttranslational modification steps [147,148,149].

Mutations affecting SOD1 can be divided into two groups: wild-type-like (WTL) mutations, the enzymatic activity of which is similar to that of the normal functional protein, and metal-binding region (MBR) mutations, which decrease the catalytic activity of the enzyme [142]. Both types of mutations were shown to result in impaired copper homeostasis in animal ALS models and the intracellular accumulation of copper [150,151]. This effect is problematic, as excessive copper acts as a trigger of oxidative stress, lipid peroxidation, apoptosis and the formation of SOD1 aggregates. Previously, mutations leading to the loss of SOD1 enzymatic activity were assumed to be involved in the pathogenesis of ALS; however, the enzymatic activity of SOD1 in many patients with SOD1-ALS is the same as that of the non-mutated protein [152].

#### 2.1.1. SOD1 and Copper(II)-Diacetyl-bis(N4-Methyl-Thiosemicarbazone (CuATSM)

The copper compound CuATSM has begun to be used as a marker of oxidative stress and hypoxia for tissue imaging using positron emission tomography, as it was shown to be selectively distributed or retained in tissues exposed to hypoxia and oxidative stress [153,154]. This copper complex is currently under study as a potential therapy for ALS, as it may have dual effects. First, CuATSM may act as a copper or even zinc carrier, allowing these two metals to bind SOD1 and thus promoting the proper catalytic function of SOD1 in SOD1-ALS; in addition, it may also exert an antioxidant effect and scavenge peroxynitrite. Thus, the therapeutic effects of CuATSM might not be limited to SOD1-ALS or ALS in general; it may also be useful for the treatment of other neurodegenerative diseases.

In 2019, the Australian researchers Farrawell et al. published a study suggesting the ability of CuATSM to deliver copper or zinc to metal-deficient SOD [155]. In a cell culture experiment in cells expressing ten different SOD1-ALS mutations, the researchers found that in the presence of WTL mutations, i.e., mutations that preserve the ability of the enzyme to bind copper or zinc, the administration of CuATSM led to the delivery and binding of copper to SOD1, accompanied by proper SOD1 maturation, increased SOD1 activity and reduced SOD1 aggregation, as well as increased cell viability. CuATSM was not found to affect mutations in the MBR, which allows the protein to bind copper or zinc. A study by Roberts et al. suggested the same mechanism of action [156]. Furthermore, McAllum et al. compared the effects of CuATSM and riluzole, the only drug commonly used to treat ALS, which inhibits glutamatergic transmission and prolongs the survival of patients by just a few months. The highest tested dose of CuATSM prolonged the survival of transgenic ALS model mice by as much as 26%, while riluzole extended survival by only 3.3% and was not associated with an improvement in motor performance [157].

Although zinc merely stabilizes the structure of SOD, the delivery of zinc to SOD1 using ZnATSM had a therapeutic effect similar to that of CuATSM. Furthermore, when ZnATSM was administered, biochemical analysis of animal spinal cord tissue showed that both the total copper level and level of copper bound to SOD1 increased, probably due to so-called transmetalation, i.e., the exchange of one metal for another [158]. Oral supplementation of small amounts of zinc might have a protective effect in SOD-ALS [159].

In contrast, Soon et al. suggested that CuATSM plays a different role, i.e., that of a peroxynitrite scavenger [160]. Peroxynitrite, formed when superoxide reacts with nitrogen oxide, is responsible for hydroxylation and nitration of the amino acid tyrosine as well as of many other proteins; consequently, it can induce the apoptosis of motor neurons. Moreover, peroxynitrite activates microglial cells and astrocytes and induces the development of neuroinflammation. Thus, peroxynitrite is involved in not only the pathogenesis of ALS but also neurodegeneration in general.

#### 2.1.2. SOD1 and the Nrf2/ARE Pathway

The Nrf2-Keap1 signalling pathway is the main regulator of the cellular response to stress caused by ROS. The activation of this pathway plays an important role in protection against the development of neurodegenerative, cardiovascular, cancerous and metabolic diseases [161,162]. The transcription factor Nrf2 controls the antioxidant response as it regulates the expression of genes encoding enzymes involved in the phase II biotransformation of foreign substances. Among the seven functional domains of Nrf2, i.e., Neh 1–7, The Neh2 domain is crucial; this domain includes two binding sites that interact with a proteinaceous Nrf2 repressor, Keap1, and thereby regulates Nrf2 stability [163,164,165].

Under basal conditions, Nrf2 is conjugated in the cytoplasm with the Keap1 protein, which controls Nrf2 degradation. However, if the cell is exposed to oxidative stress or xenobiotics, the cysteine residues of Keap1 are modified, changing the structure of Keap1 and its subsequent polyubiquitination and degradation. Therefore, the interaction between Keap1 and Nrf2 is disturbed, and Nrf2 is translocated into the nucleus, where it forms a transcription complex with Maf proteins. This complex recognizes antioxidant response element (ARE) sequences in the promoters of genes such as NAD(P)H quinone oxidoreductase 1, haem oxygenase 1, glutathione S transferase and glutamate-cysteine ligase and thus controls their expression [166].

The possible relationship between SOD1 and Nrf2 was described for the first time in 2005 by Kirby et al., who found that the Nrf2 mRNA level and expression of Nrf2-dependent genes were markedly reduced in the motor neurons of mice carrying the SOD1 (G93A) mutation [167]. Similar results were obtained by Sarlette et al., who analysed neurons in the primary motor cortices and spinal cords of patients with ALS after their death and found that the mRNA and protein expression of Nrf2 was reduced. In contrast, the mRNA expression of the Nrf2 repressor Keap1 was slightly increased in the motor cortex but not the spinal cord, and the expression of the Keap1 protein itself was not increased [168]. Similar conclusions were also reached by Pehar et al., who found that cells with reduced Nrf2 levels were more susceptible to apoptosis [169]. Somewhat different results were achieved by Kraft et al.; although the activity of Nrf2 was not increased in mutant SOD1 mice, Nr2 was active initially in the distal muscles and gradually became active in the proximal muscles, consistent with the progression of ALS from distal to proximal muscles [170]. This may reflect an adaptive response of muscles upon their exposure to an increased level of oxidative stress. By lowering the generation of oxidative stress pharmacological therapies targeting Nrf2 activation might provide a neuroprotective effect in patients with ALS. Vargas et al. generated mice expressing SOD1-G93A and found that their astrocytes also exhibited increased Nrf2 expression. This increase in expression had a protective effect against degeneration in motor neurons, where production of the antioxidant glutathione was elevated, resulting in prolonged survival of the studied animals [171].

In contrast, when Guo et al. deleted the Nrf2 gene from mice expressing SOD1-G93A, they found that the disease was only very slightly accelerated [172].

##### Modulators of the Nrf2/ARE Pathway

Nrf2 can be activated by both natural and synthetic compounds. For example, sulforaphane (SFN), found is present in Brassica vegetables such as broccoli, cauliflower, kale, cabbage and Brussel sprouts, the richest source of SFNs such as SFN glucosinolate, is one of the strongest natural activators of Nrf2 [173]. SFN can modify cysteine residues in the Keap1protein, thus releasing and activating Nrf2 [174]. The gene that encodes gamma-glutamylcysteine synthetase, which is important for the synthesis of glutathione, is activated by SFN [175,176]. Reduced glutathione (GSH) is important for the prevention of free-radical-induced damage. In cultured astrocytes, SFN increased the rate of GSH release by up to 2.4 times that with SFN administration, resulting in a decrease in the oxidative stress level in animal models of many diseases [176,177,178,179,180]. In 2018, Sedlák et al. published a study in which GSH levels in the human brain were found to be increased after seven days of SFN administration [181]. Indeed, the effect of SFN seems to depend on Nrf2 pathway activation because no neuroprotection was achieved when SFN was administered with an inhibitor of gamma-glutamylcysteine synthetase, which is necessary for the synthesis of glutathione, or when it was administered to mice with Nrf2 gene deletion [182,183,184]. Importantly, in neurons, SFN exerts a protective effect in mitochondria, on which highly metabolically active neurons depend [185,186,187,188,189,190]. SFN induces the increased expression of genes responsible for adenosine triphosphate biogenesis and prevents toxin-induced decreases in the production of adenosine triphosphate [187].

SFN seems to also have anti-inflammatory effects. It reduces the expression of tumour necrosis factor, interleukin (IL)-1β, IL-6, cyclooxygenase 2 and inducible nitric oxide synthetase and thus generally reduces neuroinflammation [179,187,188,189,190,191]. In contrast, SFN can increase expression of the cytokines IL-4 and IL-10, exerting an anti-inflammatory effect [192,193]. SFN further exerts a neuroprotective effect by increasing the expression of brain-derived neurotrophic factor, which is involved in neurogenesis and is critically important in learning and memory processes, which are often affected by neurodegenerative diseases [194]. Given that oxidative stress and neuroinflammation, both of which can be reduced by SFN, are two major factors that damage cells in neurological, particularly neurodegenerative, diseases, the use of SFN as a disease-modifying drug in the field of neurology seems very promising.

Another natural activator of Nrf2, epigallocatechin gallate (EGCG), is found in green tea. As shown by Koh et al. and Xu et al., EGCG delayed the onset of disease symptoms in an ALS mouse model [195]. Furthermore, genistein, a phytoestrogen found in soybeans, carnosine or curcumin, was shown to reduce oxidative stress through Nrf2 activation in models of several neurodegenerative diseases [196,197,198].

Among synthetic Nrf2 activators, triterpenoid compounds, as well as drugs such as bromocriptine or azathioprine, have been shown to have effects. For example, Yang et al. confirmed that the synthetic triterpenoid CDDO-methylamide exerted neuroprotective effects in models of neuroblastoma and Parkinson’s disease [199]. Furthermore, the ability of triterpenoids to activate the Nfr2/ARE pathway was investigated by Neymotin et al., who found that, compared to untreated mice, SOD1- G93A SOD1 mice treated with triterpenoids in the presymptomatic stage of ALS showed reduced weight loss, improved motor performance and prolonged survival. When treatment was started in the symptomatic stage of ALS, disease progression was slowed [200].

### 2.2. Catalase and ALS

**Figure 2:**
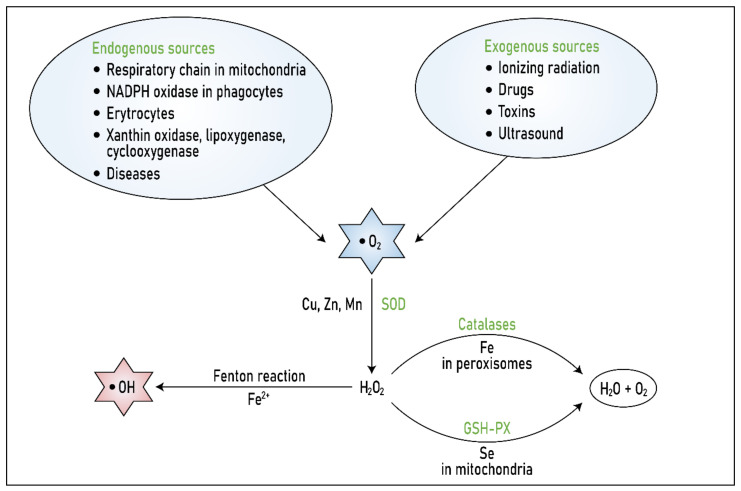
Relationship between the three major enzymes in the antioxidant protective system. Modified from [23].

Catalase, which is nearly ubiquitous in aerobic organisms, is one of the most important antioxidative enzymes. Linked to the activity of SOD1, catalase decomposes hydrogen peroxide formed through SOD1 activity into water and oxygen [201,202]. The enzyme is highly effective and can decompose millions of hydrogen peroxide molecules per second. Although highly expressed in peroxisomes, catalase is absent in mitochondria. The mitochondria of rat cardiomyocytes are the only exception. In other animal cells, GPx assumes the role of catalase [203]. The relationship between these three enzymes within the first-line antioxidant system of protection is illustrated in Figure 2.

Superoxide is produced via many endogenous processes carried out by, for example, phagocytes or the respiratory chain in mitochondria, or by the action of many enzymes, such as xanthine oxidase, which is involved in the catabolism of purines; hypoxanthines; and lipoxygenases, which catalyse the conversion of arachidonic acid to leukotrienes, important molecules in inflammatory reactions.

There are also many exogenous sources of superoxide and thus ROS, such as ionizing radiation.

Superoxide is decomposed into hydrogen peroxide by SODs with different metal co-factors and organelle localization. In the presence of iron (Fe^2+^), hydrogen peroxide is reduced, resulting in the production of the hydroxyl radical, a highly active and toxic molecule.

Therefore, SOD activity is immediately followed by catalase activity in peroxisomes and GPx activity in mitochondria; both catalase and GPx decompose hydrogen peroxide into harmless oxygen and water. These three enzymes thus contribute to first-line antioxidant protection in the body.

Three types of catalase exist: the first, containing a haem group and protoporphyrin IX, reacts with hydrogen peroxide; the second contains a haem group; and the third contains manganese instead of the haem group [204]. However, the therapeutic use of catalase is limited by its short half-life and poor ability to cross the blood–brain barrier (BBB) to enter cells, necessitating a transport system that not only prevents its degradation but also enables it to cross the BBB [205]. Singhal et al. used poly (lactic-co-glycolic acid) (PLGA) nanoparticles to deliver catalase to cultured neurons. A total of 99% of the catalase activity was preserved, catalase was successfully released from the nanoparticles and the activity of the delivered catalase was maintained for more than one month. This catalase treatment decreased the protein oxidation, DNA damage and alterations in the structure of the mitochondrial membrane, and adjusted the overall neuronal morphology [206]. Reinholz et al. administered catalase modified with putrescine to allow it better cross the BBB in transgenic fALS mice, which delayed the onset of disease symptoms [207].

### 2.3. GPxs

Among peroxidases, enzymes that catalyse the reduction of many peroxides to alcohols, are GPxs, which are selenium-dependent cytosolic enzymes that use glutathione as a co-factor. Like catalase, GPxs catalyse the conversion of hydrogen peroxide to water; during this process, GSH is oxidized to its oxidized form (GSSG). The reduction of GSSG to GSH in the reverse reaction is regulated by glutathione reductase (GR), and a physiological GSH/GSSG ratio (10:1) is maintained in cells [208]. GPxs decompose hydrogen peroxide in peroxisomes and play an important role in the protection of membranes against lipid peroxidation, which produces alcohols and water. At least eight GPxs exist in the human body. Unlike the other forms of GPx, GPx5 and GPx6 do not contain selenium; therefore, these forms of GPx probably do not effectively scavenge hydrogen peroxide [209].

#### GPX4 and Ferroptosis

GPX4 differs from other GPxs because of its substrate; GPX4 is the only GPx that can decompose phospholipid hydroperoxide and thus inhibit ferroptosis [210], a newly discovered type of cell death. Initially, only two types of cell death, i.e., programmed death (apoptosis) and non-programmed death (i.e., necrosis) were known. However, additional types of programmed death, such as autophagia, necroptosis, pyroptosis, pyronecrosis and ferroptosis, have been discovered. The induction of ferroptosis depends on the level of free iron inside cells and the related formation of ROS. Free iron atoms (Fe^2+^) can be oxidized to Fe^3+^ in the presence of hydrogen peroxide through the Fenton reaction, in which an electron is removed from peroxide, resulting in the direct production of a hydroxyl radical within the cell [211]. This induces cellular stress, cell damage by free radicals and the initiation of ferroptosis. Therefore, cells utilize specific molecules to protect themselves from the effects of free iron, among which is ferritin [212]. Ferroptosis can be induced through the inhibition of one of two key processes. The first is inhibition of the transport of cystine into cells using the cystine/glutamate transport system (Xc-), as cystine serves as a precursor for glutathione, the main cellular antioxidant, during its synthesis [206]. The second process is the inhibition of GPX4, which, as described above, protects cells from lipid peroxidation [213]. A reduction in the function or inactivation of GPX4 causes the excessive oxidation of membrane PUFAs and the formation of lipid radicals. These effects lead to the impairment and ultimately perforation of the lipid membrane structure. Iron accumulation has been observed in the spinal cords of animal models of ALS and the motor cortices of patients with ALS by MRI, which was later confirmed by histochemistry [114]. Johnson et al. found that compared to those of controls, the red blood cells of patients with ALS showed elevated levels of lipid peroxidation markers but decreased glutathione levels, accompanied by accelerated neurodegeneration [214]. Furthermore, deletion of the *GPX4* gene in a mice model resulted in the degeneration of motor neurons in the spinal cord, paralysis and death. However, supplementation with vitamin E, an inhibitor of ferroptosis, delayed the onset of paralysis and prolonged survival [215].

Substances that prevent intracellular iron accumulation, GPX4 inactivation and GSH depletion, such as vitamin E, curcumin and EGCG, consequently act as inhibitors of ferroptosis [216,217,218].

## 3. Summary

The purpose of this paper was to provide an overview of oxidative stress and its role in ALS pathogenesis, as well as the three most important enzymes involved in antioxidant protection in the body. (Semi-)metals play a necessary role in the structure and activity of these enzymes.

Increased oxidative stress is usually present in both fALS and sALS. However, the role of oxidative stress in triggering disease, especially sALS, is unclear; a large part of the population is exposed to most of the risk factors associated with increased ROS production, but only some individuals develop ALS. The development of this disease may be due to previously unknown mutations in genes encoding proteins responsible for the body’s antioxidant mechanisms.

While ROS levels are also elevated in other neurodegenerative conditions, motor neurons, which are highly metabolically active, may be particularly susceptible to oxidative damage.

Finally, we will mention innovations in the treatment that aim to reduce oxidative stress in patients and thus are neuroprotective. A drug launched on the market also acts as a free radical scavenger, more than 20 years after the introduction of riluzole treatment. It is edaravon, which was registered in 2015 for the treatment of ALS in Japan and South Korea, and 2 years later in the USA [219]. Tofersen is being studied and is administered intrathecally and targets mutated SOD. Because it acts as an antisense ologonucleotide treatment, it is the first possible gene therapy for ALS. Antisense oligonucleotides downregulate the expression of a target molecule by forming a duplex with its coding mRNA. The highest administered dose of the drug led to a reduction in the amount of mutated SOD in the cerebrospinal fluid and also to a decrease in the amount of neurofilaments, a marker of neuronal decay [220].

AMX0035, given orally, is a combination of two substances: sodium phenylbutyrate and taurursodiol. The combination of these two substances aims both to reduce the stress of the endoplasmic reticulum and also to protect the mitochondria and thus reduce ROS production [221]. However, in addition to edaravone, these are the drugs that are being studied in clinical trials and are not yet available to patients, and edaravone is not commonly available in most countries.

In general, further research is needed to develop ROS-targeted therapies to act as causal therapies, but ROS could be targeted in supportive treatments.

Based on the available data, we recommend that our patients and their family members (who may have an increased risk of ALS) consume natural antioxidants to decrease oxidative stress levels and thus try to inhibit or slow disease progression or possibly decrease the risk of developing this disease. Rather than dietary supplements, the use of sprouts produced from bio-sprouting seeds at home would be ideal, as this would guarantee that a 100% bio-product is obtained. Many types of sprouting seeds, such as broccoli sprouts, which contain sulforaphane, one of the most powerful natural activators of Nrf2, the main regulator of the cellular response to stress induced by ROS, might be consumed. However, we need additional data on the level and activity of antioxidants and, conversely, markers of oxidative stress in serum and cerebrospinal fluid in ALS patients, and the data to demonstrate which antioxidants and to what extent this situation can be favourably influenced. Eventually, we need to answer the question of how (and if at all) this will lead to a possible improvement of patients’ prognosis.

## Data Availability

No new data were created or analyzed in this study. Data sharing is not applicable to this article.
